# Plasma-Activated Water (PAW) Decontamination of Foodborne Bacteria in Shucked Oyster Meats Using a Compact Flow-Through Generator

**DOI:** 10.3390/foods14091502

**Published:** 2025-04-25

**Authors:** Phuthidhorn Thana, Dheerawan Boonyawan, Mathin Jaikua, Woranika Promsart, Athitta Rueangwong, Sunisa Ungwiwatkul, Kanyarak Prasertboonyai, Jakkrawut Maitip

**Affiliations:** 1 Faculty of Science, Energy and Environment, King Mongkut’s University of Technology North Bangkok, Rayong Campus, Rayong 21120, Thailand; phuthidhorn.t@sciee.kmutnb.ac.th (P.T.); mathin.j@sciee.kmutnb.ac.th (M.J.); woranika.p@sciee.kmutnb.ac.th (W.P.); athittarueangwong@gmail.com (A.R.); sunisa.b@sciee.kmutnb.ac.th (S.U.); kanyarak.p@sciee.kmutnb.ac.th (K.P.); 2 Plasma and Beam Physics Research Facility, Department of Physics and Materials Science, Faculty of Science, Chiang Mai University, Chiang Mai 50200, Thailand; dheerawan.b@cmu.ac.th

**Keywords:** plasma-activated water (PAW), plasma-activated water generator, home food safety, oyster decontamination

## Abstract

This study explored the effectiveness of plasma-activated water (PAW), generated by a newly developed compact generator, for decontaminating foodborne bacteria in oyster meats. The generator effectively produced PAW with antibacterial activity when the water passed through the plasma reactor in a single cycle. The temperature of the PAW produced by the developed device did not exceed 40 °C, enabling its direct application to biological tissues immediately after production and discharge from the plasma reactor. The effects of flow rates and post-discharge times on key reactive species—including hydrogen peroxide, nitrite, and nitrate—were analyzed, along with pH and temperature. Freshly produced PAW can completely inhibit both *E. coli* and *S. aureus* in vitro, with a 5-log reduction within 5 min of treatment. Application to oyster meats led to an 86.6% and 87.9% inactivation of *V. cholerae* and *V. parahaemolyticus*, respectively. These research findings indicate that PAW generated using the developed compact flow-through generator holds promise as a food safety solution for households. The fact that complete foodborne pathogen elimination was not achieved emphasizes the need for further optimization.

## 1. Introduction

The consumption of raw seafood, including blue crab, squid, salmon, and oysters, is becoming increasingly popular among Thai consumers. Oysters are highly regarded worldwide as a delicacy, and their meat is a rich source of high-quality protein, omega-3 and omega-6 fatty acids, and essential amino acids [1]. A popular Thai dish, spicy oyster salad, is usually made with raw oyster meats. However, consuming raw or undercooked oysters poses a risk of bacterial and viral infections. One such infection is vibriosis, caused by *Vibrio* bacteria. These bacteria naturally thrive in certain coastal waters and, as oysters filter water to feed, they can accumulate *Vibrio* bacteria in their tissues. The main species of concern when consuming raw oysters are *Vibrio parahaemolyticus*, *V. vulnificus*, and *V. cholerae* [2,3,4,5,6]. These bacteria can cause various illnesses, from mild gastroenteritis to severe septicemia [7]. Other pathogens found in oysters, such as *Staphylococcus aureus*, *Salmonella* spp. and *Escherichia coli*, can lead to food poisoning [8]. Infections from these bacteria can result in conditions ranging from diarrhea to more severe illnesses, such as toxic shock syndrome [9,10].

While thermal treatments can effectively inactivate pathogens, they can also negatively affect the sensory, nutritional, and functional properties of food, particularly fresh products. Therefore, methods are needed to eliminate pathogens from meat without heat while preserving nutritional value and sensory qualities.

Cold plasma processing is a promising new method for enhancing food safety while maintaining food quality. Extensive research on the decontamination of both plant- and animal-based foods has highlighted the potential of cold plasma technologies for commercial use [11,12]. Various types of atmospheric cold plasma, including corona discharge, dielectric barrier discharge (DBD), helium and argon plasma jets, and glow discharge, have been shown to have antibacterial effects [13]. Non-thermal atmospheric-pressure plasma, generated in humid air, produces reactive oxygen and nitrogen species (RONS) such as hydroxyl (OH) radicals, atomic oxygen (O), ozone (O_3_), hydrogen peroxide (H_2_O_2_), hydrogen superoxide (HO_2_), nitric oxide (NO), and nitrogen dioxide (NO_2_) [14,15,16]. These highly reactive species play a key role in eliminating contamination on food surfaces by inhibiting harmful bacteria, viruses, fungi, and even pesticide residues [17,18,19,20]. Moreover, using cold atmospheric plasma along with plasma-activated water (PAW) can help to extend the shelf life of fresh produce by inactivating foodborne pathogens [21].

Unlike fish, oysters are typically traded alive, and some species are consumed raw. This makes it difficult to apply traditional antimicrobial or antiviral processing techniques [22]. Choi et al. [23] studied the use of dielectric-barrier discharge (DBD) plasma to inactivate *E. coli* O157:H7 in fresh oysters (*Crassostrea gigas*) and achieved a 93.54% reduction in the pathogen after 60 min of treatment. Importantly, the texture and glycogen content of the treated oysters did not significantly differ from the control. Another study by Choi et al. [19] showed that DBD plasma treatment reduced human norovirus (HuNoV) by 1.05-log and 1.68-log after 30 min and 60 min, respectively, without altering the oysters’ quality (as measured in terms of pH and color). However, direct exposure to cold atmospheric plasma (CAP) is not always practical. The effectiveness of pathogen inactivation by non-thermal atmospheric plasma depends on factors such as the type of plasma source, its geometry, power delivery, gas composition, humidity, temperature, and the texture of the target. For example, uneven and rough meat surfaces can reduce pathogen inactivation efficiency due to the non-uniform distribution of reactive species [13,24].

In contrast, remote treatment using plasma-activated water (PAW) is more versatile. Water acts as a medium to prevent direct damage to the meat’s surface from charged particles, ultraviolet rays, heat, and electricity. When exposed to cold atmospheric plasma, reactive species in the gas phase interact with the water, forming new RONS in the liquid, such as OH, H_2_O_2_, HO_2_, nitrite (NO_2_^−^, nitrate (NO_3_^−^), peroxynitrite (OONO^−^), peroxynitrous acid (ONOOH), nitrous acid (HNO_2_), and nitric acid (HNO_3_) [25,26,27,28,29]. The concentration of RONS in PAW can be controlled by adjusting plasma generation parameters, such as voltage, carrier gas, temperature, pulse frequency, and treatment time [30].

While plasma-activated water (PAW) is known for its flexibility and adaptability to diverse meat characteristics, including size, shape, texture, and surface roughness [31,32,33], information on its application for foodborne pathogen inactivation in shucked oyster meats, which are popularly consumed raw in Thailand, is scarce. To address this gap, we developed a compact flow-through PAW generator which is capable of producing antibacterial PAW with a single pass of water through the plasma reactor. This study characterizes the generated PAW and evaluates its effectiveness in decontaminating foodborne pathogens from freshly shucked oysters. This research represents the initial phase in the development of a compact plasma-activated water (PAW) generator, ultimately aiming to achieve the goal of developing this PAW generator to enhance food safety in households. This device will be capable of rapidly producing PAW on demand, exhibiting efficient antimicrobial activity within short treatment times, offering user-friendly operation without the requirement of specialized expertise, ensuring safety, and maintaining cost-effectiveness.

## 2. Materials and Methods

**Figure 1:**
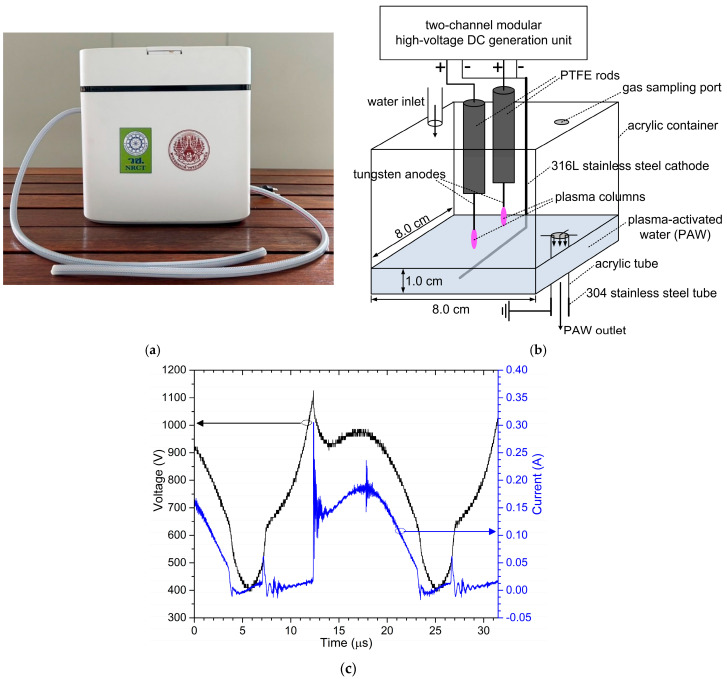
(**a**) A photograph of a compact flow-through plasma-activated water generator, (**b**) a schematic diagram of the plasma reactor used to produce plasma-activated water, which is inside the compact flow-through plasma-activated water generator, and (**c**) the voltage–current characteristics of one discharge pulse in the plasma reactor created with a 1-anode plasma discharge and a 1 cm gap.

Figure 1 illustrates the compact flow-through plasma-activated water (PAW) generator. Panel (a) displays a photograph of the complete device, which measured 31.8 cm × 15.7 cm × 31.0 cm and weighed 3.9 kg. The system incorporated a small, adjustable diaphragm water pump that drew water from an external source. The water inlet flow into the plasma reactor was designed to be continuous, and the flow rate was adjustable within the range of 100 mL/min to 500 mL/min. Panel (b) provides a schematic representation of the internal plasma reactor used for water activation. The reactor consisted of an acrylic container with dimensions of 8.0 cm × 8.0 cm × 10.0 cm. The water level in the plasma reactor was 1 cm, resulting in a water volume of 64 cm^3^ inside the plasma reactor. This container featured an inlet tube for introducing water and an outlet tube for removing the plasma-activated water. Additionally, a port was incorporated for gas measurement within the reactor, which was typically sealed during operation. The plasma-generating system comprised two tungsten anodes coated with polytetrafluoroethylene (PTFE) and positioned at the top of the container. A common cathode, constructed from 316 stainless steel and shaped into an L-configuration, was submerged in the water and rested on the container’s bottom. The discharge gap, defined as the distance between the lower ends of the anodes and the water surface, was set to 1.0 cm. The plasma-generating electrodes were connected to a compact two-channel modular high-voltage direct current (DC) generation unit, sharing a common cathode. The drainpipe was made of acrylic and was connected to a 304 stainless steel pipe, which was grounded to protect users from electric shocks caused by leakage current.

As water flowed through the internal chamber of the plasma reactor, it was activated by air plasma generated using the ambient air within the reactor. Subsequently, the plasma-activated water, containing dissolved reactive oxygen and nitrogen species, exited the plasma reactor under the influence of gravity and was discharged from the outlet of the plasma-activated water generator. The residence time of individual water fractions within the plasma activation zone, during which they were exposed to plasma and obtained reactive oxygen and nitrogen species, was determined by the water flow rate through the plasma reactor. Specifically, an increased flow rate resulted in a shorter exposure time for each water fraction to the plasma.

The electrical characteristics of the compact flow-through plasma-activated water generator were analyzed using a digital oscilloscope (Hantek DSO2C15; Qingdao Hantek Electronic Co., Ltd., Qingdao, China; 150 MHz bandwidth, 1 GSa/s sampling rate). The instantaneous discharge voltage across the electrodes, *V(t)*, was measured with a high-voltage probe (P6015A; Tektronix, Inc., Portland, OR, USA; 75 MHz bandwidth). Meanwhile, the discharge current, *I(t)*, was determined by measuring the voltage drop across a 2 Ω monitor resistor using another high-voltage probe (Hantek PP-200; Qingdao Hantek Electronic Co., Ltd., Qingdao, China; 200 MHz bandwidth). The average electrical power dissipated in the plasma discharge was calculated using the following formula:

$$\bar{P}=(1/T)\int_{0}^{T}V(t)I(t)dt$$

where *T* is the period of the voltage waveform [24].

The time-synchronized waveform of the discharge current (I) and voltage (V) for a plasma discharge in the plasma reactor generated with an anode and a discharge gap of 1 cm is shown in Figure 1c. The discharge current operated in a DC self-pulsing discharge mode with a pulse repetition rate of around 50 kHz. The pulse-average electrical power dissipated in a plasma discharge with such an anode was 76.2 W, while the power dissipated in a plasma discharge with another anode was 77.4 W. Therefore, the measured plasma power of the developed water activator was approximately 153.6 W.

Plasma-produced species within the plasma-active zone were characterized using optical emission spectroscopy (OES). A broad spectral range spectrometer (LR1-T; ASEQ instruments, Vancouver, BC, Canada) with a wavelength range of 192–887 nm was employed for general analysis. Specifically, the emission from hydroxyl (OH) radicals was examined using a high-resolution spectrometer (AvaSpec-ULS3648; Avantes, Apeldoorn, The Netherlands) with a resolution of 0.05 nm, focusing on the 265–335 nm spectral region. Optical emissions were collected approximately 2.0 cm from the plasma discharge area and averaged across the plasma volume. Each measurement consisted of an average of 5 samplings to enhance the signal quality.

### 2.1. PAW Characterizations

The experimental design in this study, which involved measuring PAW characteristics, the in vitro antimicrobial efficacy of PAW, and its effectiveness in reducing microbial contamination in shucked oysters—commonly consumed raw in signature dishes, such as spicy oyster salad—was structured to be sequential and inter-related. Optimal conditions identified in preceding experiments were utilized in subsequent investigations. For instance, the optimized water flow rate for PAW production from the developed compact flow-through PAW generator was carried forward to other subsequent experiments. This interconnected experimental design aimed to establish an appropriate method for the decontamination of shucked oysters or other food products using PAW generated by the developed device.

Reverse osmosis (RO) water was used to produce plasma-activated water (PAW) via a compact flow-through plasma-activated water generator.

In order to investigate the influence of the water flow rate through the plasma reactor and post-discharge evolution on the concentrations of long-lived reactive oxygen and nitrogen species (RONS) in PAW relevant to antimicrobial activity—particularly nitrite (NO_2_^−^), nitrate (NO_3_^−^), and hydrogen peroxide H_2_O_2_ [34,35,36]—a series of measurements were conducted under the following conditions.

For the investigation of water flow rate effects, the concentrations of nitrite, nitrate, and hydrogen peroxide in 50 mL aliquots of PAW, along with pH and temperature, were measured at water flow rates of 100, 150, 200, 250, 300, 400, and 500 mL/min immediately after PAW generation. The optimal condition was then selected based on the flow rate that was hypothesized to maximize peroxynitrite (ONOO^−^) formation via the reaction between hydrogen peroxide and nitrite [36]. The condition favoring the highest hydrogen peroxide concentration (H_2_O_2_ being the limiting precursor for ONOO^−^ formation due to its lower molecular abundance) was assumed to be the optimal condition.

For the investigation of post-discharge evolution effects, the optimal water flow rate identified in the previous part was used to investigate post-discharge evolution. The concentrations of nitrite, nitrate, and hydrogen peroxide were measured in 50 mL aliquots of PAW at storage durations of 0, 5, 10, 15, 20, 25, 30, and 60 min.

Furthermore, recognizing that the production of larger PAW volumes requires extended processing times and introduces the effect of post-discharge evolution on previously generated PAW, a study on the temporal changes in nitrite, nitrate, and hydrogen peroxide concentrations was performed during the production of PAW volumes of 0.5, 1, 2, 3, 4, 5, and 6 L. The measurements were performed immediately upon reaching each target volume.

The concentrations of hydrogen peroxide in the PAW were determined using H_2_O_2_ test strips (Quantofix^®^ Peroxide 25; Macherey-Nagel GmbH & Co. KG, Düren, North Rhine-Westphalia, Germany) with a measurement range of 0–25 mg/L. The nitrite and nitrate concentrations were quantified using Quantofix^®^ Nitrite/Nitrate test strips (Macherey-Nagel GmbH & Co. KG, Düren, North Rhine-Westphalia, Germany), which measure nitrite levels from 0 to 80 mg/L NO_2_^−^ and nitrate levels from 0 to 500 mg/L NO_3_^−^.

The physicochemical properties of the PAW, including pH and temperature, were measured using a portable, pen-type digital meter (AMT03R; Amtast USA Inc., Lakeland, FL, USA). 

### 2.2. In Vitro Assessment of the Antibacterial Activity of PAW

The experiments in this section aimed to investigate the effects of treatment time and post-discharge evolution on the antimicrobial efficacy of PAW produced by the developed compact flow-through PAW generator against foodborne pathogenic bacteria, utilizing in vitro testing. *Staphylococcus aureus* (Gram-positive) and *Escherichia coli* (Gram-negative) were chosen as representative strains.

A 50 mL volume of PAW was generated by the developed PAW generator at an optimal water flow rate of 250 mL/min. The PAW was tested under three distinct post-discharge time conditions—0 min (tested immediately after generation), 30 min, and 60 min—in order to investigate the influence of storage time on the bactericidal effect of the PAW.

Aerobic bacterial counts were determined using the method outlined in Chapter 3 of the FDA’s Bacteriological Analytical Manual (BAM). Each microorganism was prepared in sterile saline to match a 0.5 McFarland turbidity standard and was then diluted in sterile distilled water to achieve a final concentration of 10^6^ colony-forming units per milliliter (CFU/mL).

For each antimicrobial test, 1.0 mL of each bacterial suspension was added to 9.0 mL of PAW and thoroughly mixed. The mixtures were incubated for distinct treatment durations of 5 min, 10 min, and 20 min. Following incubations, 0.1 mL aliquots were spread onto plate count agar (standard methods agar) and incubated at 37 °C for 24 h.

### 2.3. Assessment of PAW’s Effectiveness in Decontaminating Freshly Shucked Oysters

The results from the experiments described in Section 2.1 and Section 2.2 indicate that the optimal conditions for the production and application of plasma-activated water (PAW), generated by the developed PAW device, included a water flow rate of 250 mL/min and the immediate use of PAW after generation and discharge. Under these conditions, the PAW exhibited the highest bactericidal efficacy against the pathogenic bacteria *Escherichia coli* and *Staphylococcus aureus* in vitro. Furthermore, it was found that a 5 min treatment was sufficient to completely inhibit both microorganisms in the in vitro setting.

However, considering that the inactivation of pathogenic bacteria in shucked oysters was more complex than in in vitro conditions, the treatment time for evaluating the efficacy of PAW generated by the developed device for inactivating foodborne pathogens in shucked oysters was set at 10 min—twice the in vitro treatment time. This 10 min duration also fell within the expected time frame proposed by the developers, which was no longer than 20 min for the effective decontamination of food materials using PAW produced by the developed device. Moreover, the short treatment duration reduces the likelihood or extent to which nitrite, nitrate, and nitrous acid dissolved in the PAW can diffuse into and accumulate within oyster tissues. These compounds are precursors that can potentially lead to the formation of nitrosamines, which are classified as carcinogenic to humans [37,38,39].

Oyster meats (*S. commercialis*) were sourced from a local seafood market in Rayong, Thailand. Bacterial enumerations (*E. coli*, *Salmonella*, *Vibrio*, and *S. aureus*) were conducted following the procedures detailed in the FDA’s Bacteriological Analytical Manual (BAM) with modification. Oyster meat samples weighing 25 g each were selected and immersed in 100 mL of PAW for 10 min. They were then transferred to 225 mL of 0.1% peptone water and homogenized using a Stomacher blender at 100 rpm for 30 s.

The oysters were immersed in sterile distilled water instead of PAW for the control condition. The total bacterial count, *S. aureus*, *Salmonella* spp., *V. cholerae*, and *V. parahaemolyticus* were analyzed using the spread plate method, while *Escherichia coli* was assessed using the most probable number (MPN) method. 

### 2.4. Statistical Analysis

All measurements were conducted in triplicate. The data were analyzed with IBM SPSS Statistics 28 software. The statistical analysis involved conducting a one-way analysis of variance (ANOVA). The significance of differences between pairs of group means was assessed using Tukey’s honestly significant difference (Tukey’s HSD) test. A pair with *p*-values less than 0.05 (*p* < 0.05) was considered statistically significant. The results were presented as mean values, with standard deviations (SDs) derived from three repetitions in each experiment.

## 3. Results

### 3.1. Plasma Diagnostics

**Figure 2:**
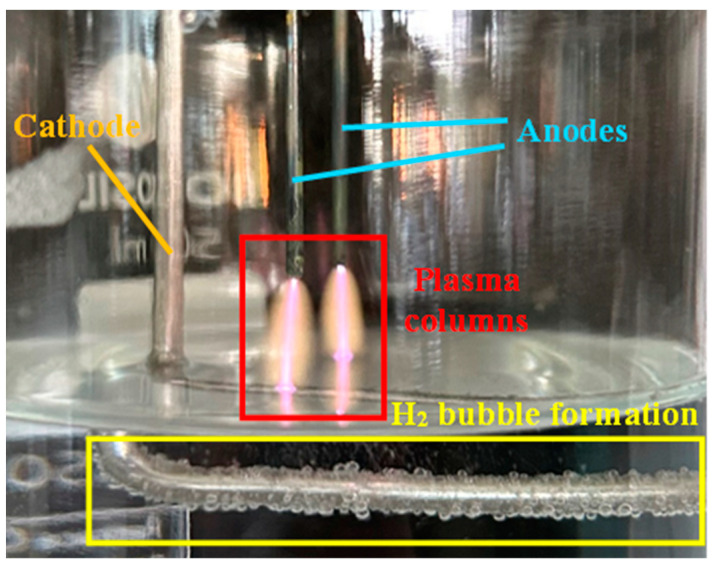
The plasma columns and hydrogen bubbles generated around the cathode in the anodic plasma electrolytic reactor of the compact flow-through plasma-activated water generator. The system features two anodes with a 1 cm discharge gap.

Figure 2 presents a photograph of plasma discharges within the reactor of our compact PAW generator. Two distinct plasma columns were observed above the water surface using a pin-to-water plane electrode configuration. The discharge gap between the pin anodes and the water surface was maintained at 1 cm. During plasma discharge, the reduction half-reaction of water electrolysis occurred. This process resulted in the production of hydrogen (H_2_) gas bubbles, readily observed around the surface of the immersed stainless steel rod cathode, confirming the reduction of water to hydrogen.

**Figure 3:**
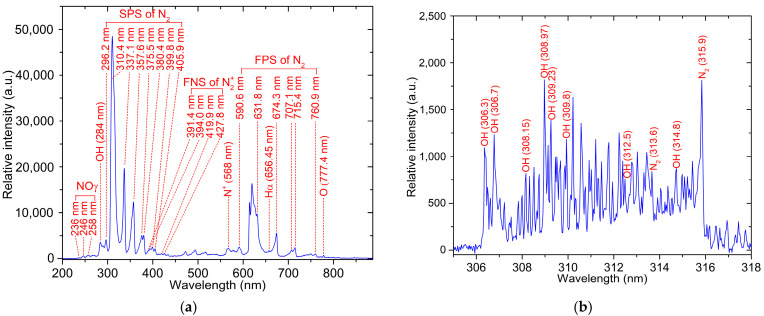
(**a**) The broadband optical emission spectrum and (**b**) the hydroxyl (OH) radical emission spectrum of the plasma generated within the reactor of the compact flow-through plasma-activated water generator.

Optical emission spectroscopy (OES) was used to monitor the species generated in the discharge area by analyzing the light in the ultraviolet, visible light, and near-infrared ranges. The characteristic light emissions from the plasma discharge within the reactor of the compact flow-through plasma-activated water generator are illustrated in Figure 3. Photons emitted from nitrogen molecules (N_2_) and nitrogen molecule ions (N_2_^+^) were observed across specific wavelength ranges, as shown in Figure 3a. The second positive system (SPS) of N_2_ (C^3^Π_u_ → B^3^Π_g_) was detected between 296.2 nm and 405.9 nm, while the first positive system (FPS) of N_2_ (B^3^Π_g_ → A^3^Σ_u_^+^) appeared between 590.6 nm and 760.9 nm [40,41]. The peaks of the first negative system (FNS) of N_2_^+^ (B^2^Σ_u_^+^ → X^2^Σ_g_^+^) were observed between 291.4 nm and 427.8 nm [41]. These observations suggest that excited nitrogen molecules are the primary species in the plasma zone. The excited nitrogen molecules (N_2_(A^3^Σᵤ⁺), N_2_(B^3^Π_g_), and N_2_ (C^3^Π_u_)), along with nitrogen molecular ions (N_2_^+^), are formed through electron impact excitation and the ionization of neutral nitrogen molecules [42,43].

Additionally, peaks around 236 nm, 246 nm, and 258 nm corresponded to light emitted from the γ-band of NO (A^2^Σ^+^ → X^2^Π) [44]. The emission spectrum also included signatures from hydroxyl radicals (OH at 284 nm), singly ionized nitrogen (N^+^ at 568 nm), atomic hydrogen (H_α_ at 656.45 nm), and atomic oxygen (O at 777.4 nm) [29,45,46,47].

Furthermore, an additional spectrum within the 600–650 nm range of the first positive system of N_2_ could be attributed to either atomic oxygen (O) [48,49] or a positively charged oxygen ion (O_2_^+^) [50,51].

Figure 3b highlights the light emission from hydroxyl (OH) radicals, which occurred between 306 nm and 312 nm. However, the OH emission within the range of 312 nm to 316 nm overlapped with emissions from N_2_ vibrational transitions, causing spectral interference [52,53].

The generation of significant species, such as hydrogen peroxide (H_2_O_2_), nitrite (NO_2_^−^), nitrate (NO_3_^−^), nitrous acid (HNO_2_), and nitric acids (HNO_3_), within plasma-activated water can be traced back to the species produced in gaseous plasma, as revealed by the emission spectroscopy. This connection will be further elaborated upon in the following discussion. The plasma-produced species generated in the gas phase can react with each other or with nitrogen, oxygen, or water molecules in the surrounding air, leading to the formation of other new species.

Plasma discharge in contact with water produces OH radicals in the gas phase. Several mechanisms contribute to the generation of these radicals. The following reactions are expected to be involved:

e + H_2_O → OH + H + e (1)

H_2_O⁺ + H_2_O → OH + H_3_O⁺ (2)

N_2_(A^3^Σ_u_^+^) + H_2_O → OH + H + N_2_ (3)

O(^1^D) + H_2_O → 2OH (4)

O^−^ + H_2_O → OH + OH^−^ (5)

H_2_O⁺ + e → OH + H (6)

H_2_O⁺ + H^−^ → OH + H_2_ (7)

H_2_O⁺ + OH^−^ → H_2_O + OH (8)

H_3_O⁺ + e → OH + H_2_ (9)

H_3_O⁺ + H^−^ → OH + H_2_ + H (10)

H + O_2_ → OH + O (11)

NO + HO_2_ → OH + NO_2_ (12)

O + H_2_ → OH + H (13)

Hydroxyl radicals are primarily formed through the dissociation of water molecules caused by direct electron collisions (Reaction (1)) [45,54]. Additional mechanisms include the dissociation of water molecules induced by the oxidaniumyl (H_2_O^+^) radical (Reaction (2)) [45,55,56], metastable molecular nitrogen N_2_(A^3^Σᵤ⁺) (Reaction (3)), excited atomic oxygen (O(^1^D)) (Reaction (4)), and negative ions of atomic oxygen (O^−^) (Reaction (5)) [33,42,43,45].

The presence of water molecules significantly influences the gas-phase plasma chemistry, particularly the concentrations of OH radicals and hydrogen peroxide (H_2_O_2_). Water molecules are transferred from the liquid surface into the plasma phase during discharge due to ion bombardment (sputtering), thermal heating (evaporation), and the emission of hydrated ions induced by the electric field [57,58,59]. Once in the plasma, water ionization produces H_2_O⁺, which further reacts to form OH radicals through various pathways, as shown in Reactions (6)–(8) [34,55,57,60]. Similarly, the hydronium ion (H_3_O⁺) contributes to OH radical formation through Reactions (9) and (10) [57]. The chemical reactions between H and O_2_, NO and HO_2_, and O and H_2_ also contribute to the production of OH, as shown in Reactions (11) to (13) [33,43,45,61].

The atomic hydrogen (H) detected by OES is a radical formed alongside OH during the dissociation of water molecules. This process occurs when water molecules collide with electrons or excited gas molecules, such as N_2_(A^3^Σ_u_^+^) [43,45]. Additionally, H is produced through the chemical reaction between OH and H_2_ [61], which occurs around the immersed cathode, as seen in Figure 2. Similarly, nascent oxygen (O) is generated through the dissociation of oxygen molecules. This dissociation is driven by collisions with electrons or excited gas molecules, such as N_2_(A^3^Σ_u_^+^), N_2_(B^3^Π_g_), N_2_ (C^3^Π_u_), and excited water molecules (H_2_O*), or through reactions between oxygen molecules and hydrogen radicals [42,43,45,62].

The recombination of hydroxyl (OH) radicals (Reaction (14)) is the primary pathway for the production of hydrogen peroxide (H_2_O_2_). This process can occur in the gas phase, liquid phase, or at the gas–liquid interface [54,63]. Another significant mechanism for H_2_O_2_ generation involves the recombination of hydroperoxyl radicals (HO_2_) (Reaction (15)) [56]. HO_2_ radicals can form through various reactions, including interactions between OH and H_2_O_2_, OH and HNO_2_, OH and ozone (O_3_), H and O_2_, H_2_O_2_ and O, O_2_ and H, and O_3_ and H [33,42,43].

OH + OH → H_2_O_2_ (14)

HO_2_ + HO_2_ → H_2_O_2_ + O_2_ (15)

Just as hydroxyl (OH) radicals are precursors in the production of hydrogen peroxide, nitric oxide (NO) and nitrogen dioxide (NO_2_) serve as precursors in the production of nitrite, nitrate, nitrous acid, and nitric acid. Several mechanisms contribute to the generation of these radicals. The possible chemical reactions involved in the generation of NO and NO_2_ are as follows [34,42,43,54,64,65,66]:

N_2_* + O → NO + N (16)

N_2_ + O → NO + N (17)

N + O_2_ → NO + O (18)

N + OH → NO + H(19)

N + O → NO(20)

N_2_(A^3^Σ_u_^+^) + NO_2_ → NO + O + N_2_ (21)

NO + O → NO(22)

2NO + O_2_ → NO_2_ (23)

NO + OH → NO_2_ + H (24)

NO + HO_2_ → NO_2_ + OH (25)

N_2_* denotes excited nitrogen molecules [65]. Atomic nitrogen (N) present in Reactions (18)–(20) could form via electron impact dissociation of nitrogen molecules or the dissociative recombination of electrons with N_2_^+^ [34,42].

The mechanisms involved in the formation of nitrous acid (HNO_2_) and nitric acid (HNO_3_) in the gas phase are presented as follows [43,56]:

NO + OH → HNO_2_ (26)

NO_2_ + HO_2_ → HNO_2_ + O_2_ (27)

NO + NO_2_ + H_2_O → 2HNO_2_ (28)

NO_2_ + OH → HNO_3_ (29)

NO + HO_2_ → HNO_3_ (30)

The formation of significant species in PAW, such as nitrite (NO_2_^−^), nitrate (NO_2_^−^), and hydrogen peroxide (H_2_O_2_), occurs primarily through the reactions at the gas–liquid interface and the dissolution of radicals from the gaseous phase into the aqueous phase. Due to their high Henry’s law solubility coefficients—approximately 9 × 10^2^ mol m^−3^ Pa^−1^ for H_2_O_2_ and 3.1 × 10^−1^ mol m^−3^ Pa^−1^ for OH—gaseous H_2_O_2_ and OH readily dissolve into PAW through the gas–liquid interface [42].

Another significant process contributing to the formation of OH radicals in plasma-activated water (PAW)—and subsequently, to the production of H_2_O_2_—involves plasma discharge generated by pin-to-water plane configuration electrodes, where the liquid serves as the cathode. This process occurs through charge transfer collisions between impinging positive ions and water molecules. When positive ions, such as the N_2_^+^, N^+^, and O^+^ produced in the plasma, collide with water molecules at the liquid surface, they transfer their charge to the water molecules, forming water cations. These water cations then react with surrounding water molecules to produce OH radicals and H_3_O^+^ ions (Reaction (2)). Notably, this process remains efficient even at low ion energies [60].

Most of the NO_2_^−^ and NO_3_^−^ present in the PAW is derived from the partial dissociation of dissolved HNO_2_ and HNO_3_ [54,67]. Since HNO_2_ and HNO_3_ also exhibit high Henry’s law solubility coefficients—approximately 4.8 × 10^−1^ mol m^−3^ Pa^−1^ and 2.1 × 10^3^ mol m^−3^ Pa^−1^, respectively—all gaseous HNO_2_ and HNO_3_ readily dissolve into PAW [42,65]. Additionally, chemical reactions at the plasma–/gas–liquid interface can contribute to their formation. The reactions between OH and NO (Reaction (26)) and OH and NO_2_ (Reaction (29)) at the interface region lead to the formation of HNO_2_ and HNO_3_, respectively, which further undergo dissolution to bulk liquid [66,67].

### 3.2. PAW Characteristics

**Figure 4:**
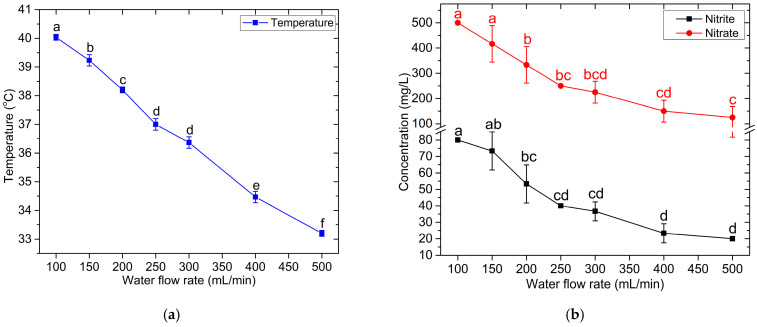

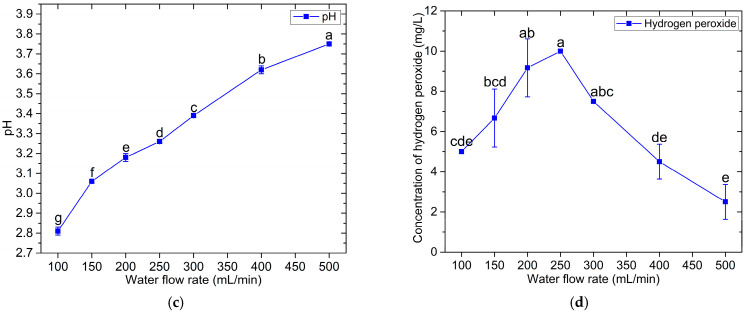
The effect of water flow rate on PAW properties: temperature (**a**), concentrations of NO_2_^−^ and NO_3_^−^ (**b**), pH (**c**), and H_2_O_2_ concentration (**d**). Error bars represent the standard deviation (SD) of the mean from triplicate measurements. Different letters above the error bars indicate significant differences at the *p* < 0.05 level (one-way ANOVA followed by Tukey’s HSD post hoc test).

Figure 4 shows the temperature and the concentrations of hydrogen peroxide, nitrite, and nitrate in the PAW generated by the compact flow-through plasma-activated water generator at different water flow rates. These measurements were performed immediately after collecting 50 mL of PAW from the outlet of the compact flow-through plasma-activated water generator. The temperature of the PAW decreased from 40.0 °C to 33.2 °C when the water flow rate through the plasma reactor increased from 100 mL/min to 500 mL/min, as shown in Figure 4a. The temperature decrease seems gradual and consistent across the flow rates. These experimental results are reasonable, as an increased flow rate would decrease the residence time of the water in the plasma reactor, resulting in a lower temperature of the PAW. The developed PAW generator can produce plasma-activated water at a temperature not exceeding 40 °C, allowing for the application of the PAW to soft matter, including biological tissues, without causing thermal damage [68].

The concentrations of NO_2_^−^ and NO_3_^−^ decreased consistently as the water flow rate increased, as shown in Figure 4b. At a flow rate of 100 mL/min, the nitrite concentration was around 80 mg/L, dropping to approximately 20.0 mg/L at 500 mL/min. Meanwhile, at 100 mL/min, the nitrate concentration was 500 mg/L, reducing to around 200.0 mg/L at 500 mL/min. The concentrations of nitrite and nitrate decreased with an increasing water flow rate, likely due to the reduced interaction time between the plasma and water. At lower flow rates, the plasma had more time to interact with the water, resulting in the higher production of nitrite and nitrate. The nitrate concentrations were consistently higher than the nitrite concentrations, suggesting that nitrate may be the dominant stable product under these conditions.

Figure 4c presents the pH values of PAW produced by the compact device using RO water with an initial pH of 6.15. The splitting of HNO_2_ and HNO_3_ in PAW not only produces NO_2_^−^ and NO_3_^−^ ions but also H^+^ and H_3_O^+^ ions [33,67], leading to a decrease in the pH of PAW, especially at low water flow rates. As the flow rate increased, the pH value gradually increased. The observed increase in the PAW pH with an increasing water flow rate is likely attributed to the reduced residence time of the water within the plasma reactor at higher flow rates. This shorter exposure duration led to a diminished dissolution of acidic species, particularly HNO_2_ and HNO_3_, from the plasma gas phase into the water. Consequently, the concentration of H^+^ or H_3_O⁺ ions in the PAW was lower, resulting in a higher pH.

The concentration of H_2_O_2_ increased from 5.0 mg/L at a water flow rate of 100 mL/min to 10.0 mg/L at 250 mg/L and then decreased as the flow rate increased, as shown in Figure 4d. At 500 mL/min, the concentration dropped significantly to around 2.5 mg/L. The H_2_O_2_ concentration of the PAW at low flow rates exhibited different characteristics compared to nitrite and nitrate. This may be attributed to the various chemical reactions in activated water, where H_2_O_2_ is consumed. Furthermore, the maximum molarity of the H_2_O_2_ was only 0.29 mM (or 10 mg/L at a water flow rate of 250 mL/min), whereas the NO_2_^−^ and NO_3_^−^ reached maximum concentrations of 1.74 mM (or 80.0 mg/L at a water flow rate of 250 mL/min) and 8.06 mM (or 500.0 mg/L at a water flow rate of 250 mL/min), respectively. The chemical reactions involved in the loss of H_2_O_2_ from the PAW in the plasma reactor are as follows [33,34,42,56,67,69,70]:

H_2_O_2_ → 2OH (31)

H_2_O_2_ + OH → HO_2_ + H_2_O (32)

H_2_O_2_ + H → OH + H_2_O (33)

H_2_O_2_ + NO_2_^−^ + H^+^ → ONOOH + H_2_O (34)

H_2_O_2_ + 4HNO_3_ → O_2_ + 4NO_2_ + 3H_2_O (35)

H_2_O_2_ + ONOOH → O_2_NOOH + H_2_O (36)

Under acidic conditions, the decreases in H_2_O_2_ or NO_2_^−^ concentrations, or both, have been reported to result from the combination of both radicals to form peroxynitrous acid (ONOOH), as shown in Reaction (34) [70,71,72,73,74,75]. Additionally, the amount of H_2_O_2_ can decrease upon reaction with HNO_3_, as shown in Reaction (35) [69], and with ONOOH to form peroxynitric acid (O_2_NOOH) (Reaction (36)) [70]. Reactions (34) and (35) show that under low flow rate conditions, which favor the formation of nitrite, nitrate, nitrous acid, and nitric acid in PAW, a significant amount of H_2_O_2_ is consumed in these reactions, leading to a lower concentration of H_2_O_2_ in the PAW. Moreover, under highly acidic conditions (pH < 3.5), the reactive species within the PAW exhibit instability and readily engage in mutual reactions, leading to a fluctuating concentration of these components [36].

In order to determine the optimal water flow conditions for plasma-activated water (PAW) production using a developed compact flow-through plasma-activated water device, three criteria were considered. First, the flow rate needs to ensure that the PAW temperature does not exceed 40 °C to allow for immediate application to biological tissues after generation (lower temperatures were preferred). Second, the conditions have to maximize H_2_O_2_ generation, as H_2_O_2_ is a precursor for peroxynitrite (ONOO^−^) [36], which has been recognized for its crucial role in antimicrobial activity [34,35,36] and dictates the amount of peroxynitrite formed from the reaction between H_2_O_2_ and NO_2_^−^ since the H_2_O_2_ concentration in the PAW is lower than that of nitrite. Third, the selected condition should minimize the generation of nitrate, nitrite, and HNO_2_ in the PAW due to concerns regarding their potential diffusion and accumulation in shucked oyster tissues [37,38], possibly leading to the formation of nitrosamines, which have carcinogenic potential [39]. Based on these three criteria, water flow rates of 250 mg/L or 300 mg/L were identified as potentially suitable for PAW production. However, since the mean H_2_O_2_ concentration was higher at 250 mg/L, this water flow rate was chosen for all subsequent experiments.

**Figure 5:**
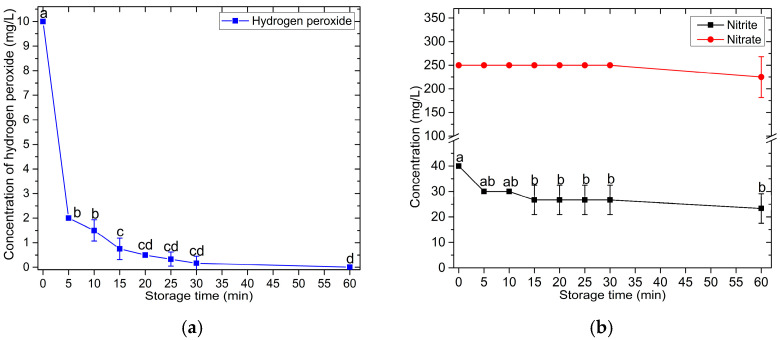
The evolution of the concentrations of H_2_O_2_ (**a**), NO_2_^−^, and NO_3_^−^ (**b**) during PAW storage at room temperature. The PAW was generated with a water flow rate of 250 mL/min. A volume of 50 mL of PAW was used to analyze the concentrations of these reactive species. The error bars represent the standard deviation (SD) of the mean from triplicate measurements. The different letters above the error bars indicate significant differences at the *p* < 0.05 level (one-way ANOVA followed by Tukey’s HSD post hoc test).

The post-discharge evolution of H_2_O_2_, NO_2_^−^, and NO_3_^−^ in the PAW after generation by the compact flow-through PAW generator was studied under different storage conditions. The volume of 50 mL of PAW used in the investigation was generated with a water flow rate of 250 mL/min. Figure 5a reveals that the concentration of H_2_O_2_ in the PAW decreased rapidly from 10 mg/L to 2 mg/L during the first 5 min of storage time and gradually declined until it became undetectable after leaving the PAW for 1 h. The post-discharge evolution of NO_2_^−^ concentration exhibited the same pattern as H_2_O_2_, as displayed in Figure 5b. The NO_2_^−^ concentration decreased from 40 mg/L to 30 mg/L within the first 5 min of storage time and then declined slightly as the storage time increased. Meanwhile, the concentration of NO_3_^−^ remained stable during the first 30 min of post-discharge time and slightly decreased when measured after 1 h of post-discharge time, as shown in Figure 5b. The decrease in H_2_O_2_ and NO_2_^−^ in this scenario is attributed to the combination of H_2_O_2_ with NO_2_^−^ under acidic conditions, forming ONOOH [71,74]. This is particularly evident in cases where the initial concentration of H_2_O_2_ is significantly lower than the initial concentration of NO_2_^−^, leading to a rapid reduction in the amount of H_2_O_2_.

In addition to reacting with H_2_O_2_, there are other mechanisms that can decrease the concentration of NO_2_^−^ in PAW. For example, NO_2_^−^ can combine with H^+^ to form NO and NO_2_ [33], both of which have very low Henry’s law solubility coefficients (1.9 × 10^−5^ mol m^−3^ Pa^−1^ for NO and 1.2 × 10^−4^ mol m^−3^ Pa^−1^ for NO_2_), causing a portion of these radicals to volatilize into the atmosphere [42,67]. The photolysis of nitrite and nitrate also contributes to the reduction of these radicals in PAW [76,77,78,79].

**Figure 6:**
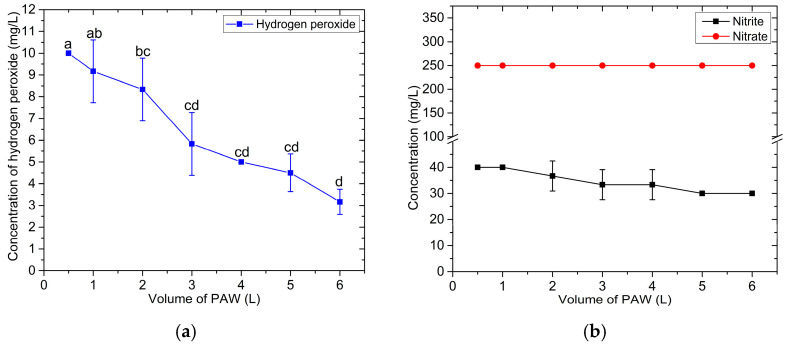
The average concentrations of H_2_O_2_ (**a**), NO_2_^−^, and NO_3_^−^ (**b**) in PAW as a function of the volume of PAW produced per batch. The error bars represent the standard deviation (SD) of the mean from triplicate measurements. The different letters above the error bars indicate significant differences at the *p* < 0.05 level (one-way ANOVA followed by Tukey’s HSD post hoc test).

Due to the limited lifetime of H_2_O_2_ caused by the reaction with NO_2_^−^ in PAW produced by the developed compact flow-through PAW generator system, the effect of the volume of PAW produced per batch on the average H_2_O_2_ concentration in PAW was investigated. In this experiment, the flow rate of water through the system was maintained at 250 mL/min, and the H_2_O_2_ concentration was measured immediately upon reaching the desired volume of PAW. The experimental results are shown in Figure 6. When the desired PAW volume per batch was increased, the average concentrations of H_2_O_2_ and NO_2_^−^ decreased. In contrast, the NO_3_^−^ concentration remained constant at 250 mg/L, regardless of the batch volume. The average concentrations of these reactive species in the reservoir resulted from the interplay between their consumption—due to reactions among them—and their replenishment by the plasma reactor. Producing larger PAW volumes requires a longer production time, which extends the post-discharge period in the reservoir. As a result, H_2_O_2_ and NO_2_^−^ undergo greater depletion, leading to lower average concentrations.

Based on the findings on storage time, production volume, and PAW temperature measurements, it is concluded that for optimal performance, PAW produced by the compact flow-through PAW generator should be used immediately after production. Alternatively, samples requiring PAW treatment should be treated directly from the generator outlet.

### 3.3. In Vitro Evaluation of Bactericidal Activity of PAW

Based on the findings in Section 3.1, which characterized the properties of PAW generated by the compact flow-through plasma-activated water generator developed in this study, we found that the optimal water flow rate for PAW production was 250 mL/min. It was also observed that the concentrations of reactive oxygen and nitrogen species (RONS), particularly hydrogen peroxide (H_2_O_2_), exhibited a clear post-discharge evolution, as shown in Figure 5a. Specifically, the concentration of H_2_O_2_ in the PAW was highest immediately after production and then gradually declined, approaching zero after 30 min of storage. Therefore, we selected PAW samples with storage times of 0 min, 30 min, and 60 min to represent PAW with the highest H_2_O_2_ concentration, PAW with nearly depleted H_2_O_2_ levels, and PAW with no detectable H_2_O_2_, respectively. These samples were used to investigate the effects of post-discharge evolution on the antimicrobial efficacy of PAW produced by the developed generator. In addition, this section also investigated the effect of treatment time on the antimicrobial efficacy of PAW produced by the developed generator in inactivating foodborne pathogenic microorganisms.

Table 1 presents the bactericidal efficacy of PAW stored for 0, 30, or 60 min against *E. coli* and *S. aureus* at contact times of 5, 10, and 20 min. The PAW was generated at a water flow rate of 250 mL/min, and a 50 mL volume was used for the evaluation.

PAW with different storage times—0, 30, and 60 min—was equally effective in inactivating *E. coli* with an initial concentration of (4.93 ± 0.75) × 10^5^ CFU/mL within 5 min of treatment. In the in vitro assessment of *S. aureus* inactivation by PAW, it was found that exposure to PAW for 5 min resulted in a significant reduction in the bacterial population. The antimicrobial efficacy of PAW against *S. aureus* increased with longer treatment durations. When comparing the bactericidal effectiveness of PAW with different storage times, the results clearly demonstrate that PAW with a storage time of 0 min—i.e., PAW used immediately after generation—exhibited significantly higher antimicrobial activity against *S. aureus* than PAW stored for 30 or 60 min. PAW that was used immediately after production was able to completely inactivate *S. aureus* with an initial concentration of (2.10 ± 0.15) × 10^5^ CFU/mL within 5 min of treatment time. PAW with a post-discharge time of 30 min was able to completely inactivate this microorganism with a plasma treatment time of 20 min. In comparison, PAW with a post-discharge time of 60 min achieved a maximum reduction of 99.8% in the quantity of this micro-organism with a plasma treatment time of 20 min. These findings suggest that the PAW produced by the developed generator should be applied immediately after production to achieve maximal microbial inactivation efficiency in food materials treated with PAW.

The observed differences in PAW efficacy against *E. coli* and *S. aureus* may be attributed to the fact that Gram-negative bacteria exhibited greater susceptibility to PAW than Gram-positive bacteria [80].

The varying bactericidal effects of PAW with different storage times against *S. aureus* were consistent with the observed instability of reactive oxygen and nitrogen species (RONS), particularly H_2_O_2_ and NO_2_^−^, in PAW. This was a result of post-discharge evolution, as demonstrated by the experimental results shown in Figure 5 and discussed in Section 3.2.

A 50 mL volume of PAW that was used for microbial inactivation immediately after being generated and discharged from the developed PAW production device contained H_2_O_2_ and NO_2_^−^ concentrations of 10 mg/L and 40 mg/L, respectively, and exhibited acidic properties with a pH value of 3.26. The bactericidal activity of this PAW was likely attributed to the effects of peroxynitrous acid (ONOOH)/peroxynitrite (ONOO^−^), as well as other radicals generated from this species, such as hydroxyl (OH) radical, peroxynitric acid (O_2_NOOH), and singlet oxygen (^1^O_2_) [34,64,70,71,72,81,82,83]. ONOOH, which is generated from the chemical reaction between NO_2_^−^ and H_2_O_2_ under acidic conditions (Reaction (34)), can decompose into ONOO^−^, which can induce bacterial cell death by both cellular apoptosis and necrosis [34,81]. ONOO^−^ can cause the lipid peroxidation of microbial membranes [82]. It can also diffuse into cells, accumulate intracellularly, and cause internal cell damage, such as DNA damage, lipid peroxidation, protein oxidation, protein nitration, and inactivation of enzymes, ultimately leading to bacterial cell death [34,64,83]. In addition to the effects of ONOOH/ONOO^−^, it was found that the sole nitrous acid (HNO_2_) solution exhibited antimicrobial activity, such as against *E. coli*, *Pseudomonas aeruginosa*, and *Saccharomyces cerevisiae* [71,84,85,86]. HNO_2_ destroys microbial cells by disrupting metabolic pathways, inhibiting respiration, interfering with DNA replication, transcription, and translation, and inducing oxidative stress, ultimately leading to cell death [84,87,88]. The relative amounts of nitrous acid (HNO_2_) and its nitrite ion (NO_2_^−^) in the solution are determined by the pH, following the equilibrium: HNO_2_ ⇌ H⁺ + NO_2_^−^. In acidic conditions (pH 2–5), both the HNO_2_ and NO_2_^−^ forms are present, with higher concentrations of HNO_2_ observed at lower pH values [89].

In the cases of PAW with post-discharge times of 30 and 60 min, where H_2_O_2_ is nearly or entirely depleted, along with other short-lived radicals generated during post-discharge reactions (such as ONOO^−^, OH, and ^1^O_2_, which have a half-life of less than 1 s [90]), only stable NO_2_^−^, NO_3_^−^, HNO_2_, and HNO_3_ remain. The antimicrobial effect of PAW observed with post-discharge durations of 30 min and 60 min was likely attributed to the presence of HNO_2_, which correlates with the detection of NO_2_^−^ in acidic PAW. The residual nitrite concentration in the PAW was approximately 26 mg/L after 30 min of storage.

### 3.4. Decontamination Efficacy of PAW on Freshly Shucked Oysters

The PAW generated from the developed compact flow-through PAW generator was evaluated for its effectiveness in reducing microbial contamination in freshly shucked oysters. The targeted microorganisms were foodborne pathogens commonly found in raw seafood, such as fish, shrimp, squid, shellfish, and sashimi, based on the standards of the Ministry of Public Health, Thailand. These standards include the total bacterial count (must be less than 100,000 CFU/g), *E. coli* (must be less than 3 MPN/g), *S. aureus* (must be less than 100 CFU/g), and the absence of *Salmonella* spp., *V. cholerae*, and *V. parahaemolyticus.*

**Figure 7:**
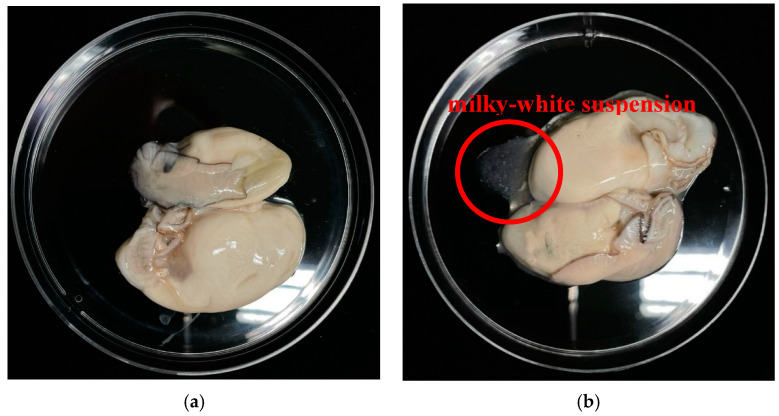
Photographs of the untreated group (**a**) and PAW-treated group (**b**) of freshly shucked oysters (*S. commercialis*) used to assess the efficacy of PAW in decontaminating foodborne pathogens.

The comparison of the physical characteristics of oysters between the untreated group and the PAW-treated group for 10 min is shown in Figure 7. Meanwhile, the percentage reduction in foodborne pathogens in oysters after PAW treatment is presented in Table 2. PAW reduced the total bacterial count by 51% and decreased the levels of *E. coli*, *S. aureus*, *Salmonella* spp., *V. cholerae*, and *V. parahaemolyticus* by 80.0%, 64.6%, 65.6%, 86.6%, and 87.9%, respectively. However, the remaining levels of total bacteria, *Salmonella* spp., *V. cholerae*, and *V. parahaemolyticus* did not meet the standards of the Ministry of Public Health of Thailand.

During the immersion of oysters in the PAW, a milky-white suspension was observed being expelled from the oysters, as shown in Figure 7b. This phenomenon may have affected the efficiency of the PAW in microbial inactivation, as components in this suspension could have interacted with and reacted with the reactive species responsible for inactivation, such as H_2_O_2_, NO_2_^−^, HNO_2_, ONOOH, ONOO^−^, OH, and ^1^O_2_, thereby reducing the number of these radicals available to function in microbial disinfection. Furthermore, the accumulation of bacteria within the tissues of oysters, particularly in their gastrointestinal tract [8,91,92], may limit the accessibility of reactive species to these areas, thereby reducing the effectiveness of PAW in disinfecting microorganisms within the oysters.

Oysters sold in fresh markets across various regions of Thailand are predominantly distributed in shucked form, packaged in containers and ready for consumption. Consumers who favor oysters typically prefer to eat them raw, for example, in dishes such as spicy oyster salad. In this study, we aimed to utilize plasma-activated water (PAW) generated by a compact flow-through plasma-activated water generator developed in-house to reduce foodborne bacterial contamination in shucked oysters. The findings indicate that PAW produced by the developed device exhibited significant potential in reducing pathogenic bacterial loads in shucked oyster samples while maintaining the quality of the oyster meat.

Future investigations will focus on optimizing the treatment process to enhance the efficacy of pathogen reduction in shucked oysters using the developed PAW system. Strategies may include increasing the number of treatment cycles or integrating PAW application with other decontamination techniques such as sonication or ultrafine bubble technology. Additionally, the developed PAW generator may be employed to stimulate seawater for use as a depuration medium in static depuration systems aimed at reducing microbial contamination in live oysters [93].

Moreover, it is essential to assess the food safety of plasma-treated shucked oysters, as PAW contains dissolved nitrite, nitrate, and nitrous acid—compounds that may accumulate in oyster tissues and potentially lead to the formation of nitrosamines, which are classified as human carcinogens [37,38,39]. Finally, the safety of the developed PAW generator for operators must be evaluated, along with an economic analysis of its application for enhancing food safety in household-level oyster processing.

## 4. Conclusions

In summary, the PAW produced by the compact flow-through plasma-activated water (PAW) generator exhibited strong antimicrobial properties against foodborne pathogens, including *E. coli*, *S. aureus*, *Salmonella* spp., *V. cholerae*, and *V. parahaemolyticus*. The bactericidal effect was primarily attributed to reactive oxygen and nitrogen species (RONS) present in PAW, such as hydrogen peroxide (H_2_O_2_), nitrite (NO_2_^−^), peroxynitrous acid (ONOOH), and nitrous acid (HNO_2_). The key findings demonstrate that PAW effectively reduced the microbial contamination levels in fresh oysters. However, further optimization of treatment time and protocols is necessary to achieve complete compliance with food safety standards. Additionally, the stability of PAW over time was examined, highlighting the importance of immediate use after generation for maximum efficacy. Overall, this research provides a promising and user-friendly solution for enhancing home food safety without relying on chemical preservatives or thermal treatments. Future studies should focus on improving PAW generation efficiency, assessing its long-term safety in food applications, evaluating user safety, such as electrical safety, and expanding its use to other perishable food items.

## Figures and Tables

**Table 1:** The antibacterial activity of plasma-activated water (PAW) stored for 0, 30, or 60 min against *Escherichia coli* (*E. coli*) and *Staphylococcus aureus* (*S. aureus*) at contact times of 5, 10, and 20 min. The PAW was produced at a water flow rate of 250 mL/min; 50 mL was used per assay. Statistical significance (*p* < 0.05) between contact times for a given storage duration is indicated by different uppercase letters. Statistical significance (*p* < 0.05) between storage durations for a given treatment time is indicated by different lowercase letters.

Bacteria	Treatment Time (min)	Number of Viable Bacterial Cells (CFU/mL)		
		0 min of Storage Time	30 min of Storage Time	60 min of Storage Time
*E. coli*	0	(4.93 ± 0.75) × 10^5 A^		
	5	0 ^B^	0 ^B^	0 ^B^
	10	0 ^B^	0 ^B^	0 ^B^
	20	0 ^B^	0 ^B^	0 ^B^
*S. aureus*	0	(2.10 ± 0.20) × 10^5 A^		
	5	0 ^B, b^	(4.73 ± 0.50) × 10^3 B, a^	(5.27 ± 0.47) × 10^3 B, a^
	10	0 ^B, c^	(1.60 ± 0.30) × 10^3 B, b^	(3.83 ± 0.21) × 10^3 B, a^
	20	0 ^B, b^	0 ^B, b^	(5.00 ± 2.00) × 10^2 B, a^

**Table 2:** The percentage reduction in food pathogens in freshly shucked oysters after 10 min of PAW treatment. The PAW was generated with a water flow rate of 250 mL/min. A volume of 100 mL of PAW was used immediately after generation to evaluate the antibacterial effect. Data with different superscript letters within the same row are significantly different at the 0.05 level (*p* < 0.05).

Microorganism	Number of Viable Bacterial Cells		Percentage Reduction (%)
	Untreated	PAW-Treated	
Total bacterial count (CFU/g)	(4.14 ± 0.26) × 10^5 a^	(2.03 ± 0.38) × 10^5 b^	51.0
*E. coli* (MPN/g)	<3	<3	-
*S. aureus* (CFU/g)	5.65 ± 0.82 ^a^	2.00 ± 1.74 ^b^	64.6
*Salmonella* spp. (CFU/g)	0.32 ± 0.04 ^a^	0.11 ± 0.06 ^b^	65.6
*V. cholerae* (CFU/g)	2.17 ± 0.30 ^a^	0.29 ± 0.13 ^b^	86.6
*V. parahaemolyticus* (CFU/g)	1.07 ± 0.23 ^a^	0.13 ± 0.23 ^b^	87.9

## Data Availability

The data that support the findings of this study are available from the corresponding author upon reasonable request.
